# Supplementary material for: Relationship Between Vitamin D Levels with In-Hospital Complications and Morphofunctional Recovery in a Cohort of Patients After Severe COVID-19 Across Different Obesity Phenotypes
Source: Nutrients. 2024 Dec 30;17(1):110. doi: 10.3390/nu17010110 (PMC11722648; doi:10.3390/nu17010110)

SUPPLEMENTARY

Table S1. Changes (Δ) in morphofunctional parameters at six months.

	Hospital discharge *N=94*	Six months *N=80*	Δ *N = 80*	*p* value
PhA	4.9±1.1	5.3±1.1	0.6±0.6	**<0.001***
SPhA	-1.1±1.1	-0.7±1.1	0.6±0.6	**<0.001***
TBW/FFM (%)	77±4.1	75±3.5	-1.1±3.7	**0.003***
FM (%)	33±8.8	32±8.7	-1.7±4.1	**0.004***
FFM (kg)	59±12	60±11	1.3±5.0	**<0.001***
FFMI (kg/m²)	20±3.2	21±3.1	1.2±1.7	**<0.001***
BCM (kg)	28±8.9	29±4.7	3.5±4.6	**<0.001***
BCM/h (kg/m)	16±4.8	18±4.55	2.2±2.6	**<0.001***
Na/K ratio	1.2±0.2	1.1±0.3	-0.1±0.5	**<0.001***
ASMM (kg)	24±5.9	24±5.6	1.2±2.1	**0.006***
ASMMI (kg/m²)	7.4±1.9	7.9±1.6	0.3±0.7	0.09
SMM (kg)	28±6.9	29±6.4	1.3±3.3	**0.001***
SMI (kg/m²)	9.7±1.9	9.9±1.9	0.4±1.2	**0.001***
SMM/w	32±5.6	32±5.1	0.5±2.5	0.19
R-HGS (kg)	23±11	27±8.0	5.6±7.1	**<0.001***
L-HGS (kg)	21±12	25±10	6.1±6.3	**<0.001***
UAG (s)	9.4±3.0	7.5±1.9	-1.9±2.2	**<0.001***
6MWT (m)	365±57	476±5.1	116±52	**0.006***
Barthel scale	92±17	99±0.2	6.1±2.5	**<0.001***
Albumin (mg/dL)	3.1±0.9	3.9±0.4	0.9±0.6	**<0.001***
Prealbumin (g/dL)	24±5.2	27±6.0	3.0±11	0.22
CPR (mg/dL)	27±54	7.6±9.4	-24±31	**0.005***
HbA1c (%)	6.2±1.1	6.5±1.2	-0.01±1.0	0.88
Vitamin D (ng/ml)	21±8.9	32±10	14±8.9	**<0.001***

Data are expressed as mean ± standard deviations. Groups were divided according to the time of evaluation. Abbreviations: PhA: phase angle; SPhA: Standardized phase angle; TBW: total body water; FFM: fat free mass; BCM: body cell mass; BCM/h: Standardized body cell mass; BMI: Body mass index; FM: fat mass; ASMM: appendicular skeletal muscle mass; SMM: skeletal muscle mass; SMM/w: skeletal muscle mass/weight; SMI: skeletal mass index; ASMMI: appendicular skeletal muscle mass index; FM: Fat mass; CRP: C reactive protein; HbA1c: glycated hemoglobin. Asterisk indicates significant difference between groups, according to Student's T-test (or Mann-Whitney test) (*p<0.05).

Table S2. Morphofunctional parameters at 6 months, according to vitamin D levels.

	All *n=80*	Vitamina D ≤ 20 ng/dL *n=8*	Vitamina D 20-29.99 ng/dL *n=28*	Vitamina D ≥30 ng/dL *n=44*	*p* value
PhA	5.3±1.1	5.12±1.2	5.20±1.2	26.3±1.1	0.083
SPhA	-0.7±1.1	-0.76±1.2	-0.72±1.2	-0.43±1.2	0.078
TBW/FFM (%)	75±3.5	76.6±4.1	73.4±4-2	73.1±3.5	0.534
FM (%)	32±8.7	37.3±7.2	30.83±8.3	29.9±8.7	0.173
FFM (kg)	60±11	59.5±12.0	59.9±11.2	61.1±11.0	0.567
FFMI (kg/m²)	21±3.1	20.5±3.2	21.1±3.8	22.2±3.1	0.653
BCM (kg)	29±4.7	29.1±8.2	29.9±8.4	30.2±4.9	0.740
BCM/h (kg/m)	18±4.5	17.0±4.3	18.5±4.3	19.5±4.6	0.777

	All<br><br>*n=80*	Vitamina D ≤ 20 ng/dL<br>*n=8*	Vitamina D 20-29.99 ng/dL<br>*n=28*	Vitamina D ≥30 ng/dL<br>*n=44*	*p* value
Na/K ratio	1.1±0.3	1.1±0.2	1.2±0.2	0.8±0.3	0.36
ASMM (kg)	24±5.6	22±5.2	24±3.9	24±5.1	0.25
ASMMI (kg/m^2)	7.4±1.9	7.3±1.6	7.5±1.7	7.5±12	0.68
SMM (kg)	25±4.3	27±6.6	29±6.1	29±4.5	0.57
SMI (kg/m^2)	8.7±1.9	9.8±1.9	9.9±2.1	10±1.9	0.53
SMM/w	32±5.1	28±5.6	33±7.1	35±5.1	0.29
R-HGS (kg)	27±8.0	26±11	27±10	29±8.1	0.19
L-HGS (kg)	25±10	25±9.2	27±8.2	29±10	0.29
UAG (s)	7.5±1.9	8.3±3.2	7.8±1.4	6.2±1.9	0.29
6MWT (m)	476±5.1	429±57	467±45	479±5.7	0.36
Barthel	99±0.2	92±10	97±4.5	99±0.2	0.51
Albumin (mg/dL)	3.9±0.4	3.9±0.9	4.0±1.0	3.9±1.4	0.50
Prealbumin (g/dL)	27±6.0	24±5.2	27±5.4	28±6.1	0.30
CPR (mg/dL)	7.6±9.4	14±27	24±5.5	6.6±5.4	0.41
HbA1c (%)	6.5±1.2	6.7±1.1	6.0±1.1	5.7±1.2	0.47
Vitamin D (ng/dL)	32±10	16±2.4	26±4.1	41±11	**<0.001**

Data are expressed as mean ± standard deviations or percentage. Groups were divided according to vitamin D levels. Abbreviations: PhA: phase angle; SPhA: Standardized phase angle; TBW: total body water; FFM: fat free mass; BCM: body cell mass; BCM/h: Standardized body cell mass; BMI: Body mass index; FM: fat mass; ASMM: appendicular skeletal muscle mass; SMM: skeletal muscle mass; SMM/w: skeletal muscle mass/weight; SMI: skeletal mass index; ASMMI: appendicular skeletal muscle mass index; FM: Fat mass; CRP: C reactive protein; HbA1c: glycated hemoglobin. Asterisk indicates significant difference between groups, according to ANOVA (or Kruskal-Wallis U test) and Tukey's multiple comparison test when required (*$p<0.05$).

Figure S1. PostCOVID19 physiotherapeutic exercises. Leaflet delivered to the patients included in our study who underwent the specific rehabilitation program. Rehabilitation and Physical Medicine Service. Virgen de la Victoria University Hospital.

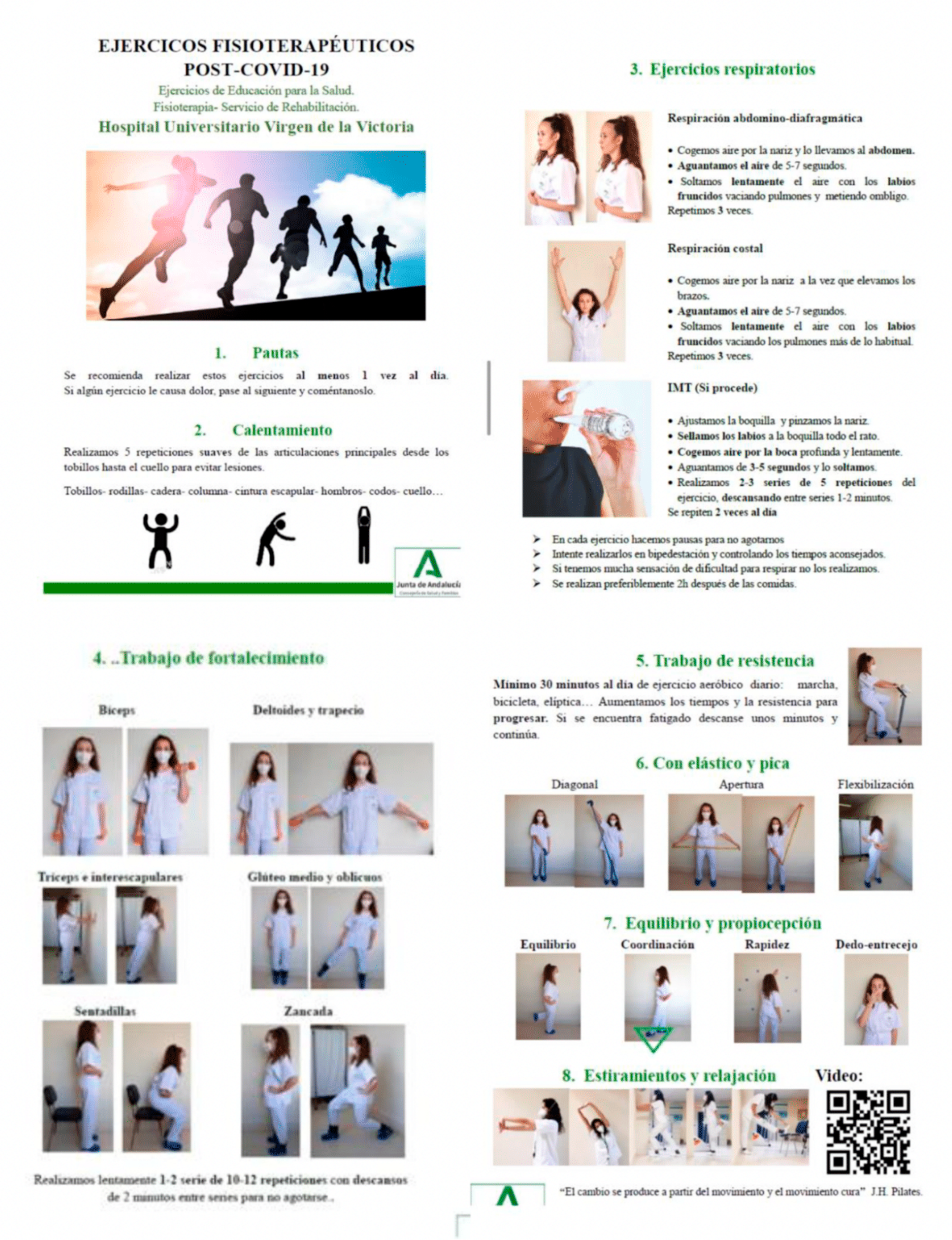

Supplement: Supplementary file 1 [file nutrients-17-00110-s001.zip › nutrients-3384211-supplementary.pdf]
